# Evaluating the Use of Telepractice for Bottle-Feeding Assessments

**DOI:** 10.3390/children8110989

**Published:** 2021-11-01

**Authors:** Madeline Raatz, Elizabeth C. Ward, Jeanne Marshall, Clare L. Burns

**Affiliations:** 1 Speech Pathology Department, Queensland Children’s Hospital, Brisbane, QLD 4101, Australia; jeanne.marshall@health.qld.gov.au; 2 School of Health & Rehabilitation Sciences, The University of Queensland, Brisbane, QLD 4072, Australia; liz.ward@uq.edu.au (E.C.W.); clare.burns@health.qld.gov.au (C.L.B.); 3 Centre for Functioning and Health Research (CFAHR), Metro South Hospital and Health Service, Brisbane, QLD 4102, Australia; 4 Speech Pathology and Audiology Department, Royal Brisbane and Women’s Hospital, Brisbane, QLD 4029, Australia

**Keywords:** pediatric, infant, pediatric feeding disorder, dysphagia, assessment, bottle-feeding, speech pathology, telepractice

## Abstract

There is currently limited evidence supporting the use of telepractice to conduct bottle-feeding assessments. This study aimed to investigate the inter-rater reliability of bottle-feeding assessments conducted via synchronous telepractice (real-time videoconferencing). Secondary aims were to investigate parent and clinician satisfaction. Bottle-feeding skills of 30 children (aged 1 month–2 years) were simultaneously assessed by a telepractice SP (T-SP) at a remote location and an in-person SP (IP-SP) at the family home. A purpose-designed assessment form was used to evaluate: (1) developmental level (screen only), (2) state, color, and respiration, (3) oral motor skills, (4), infant oral reflexes, (5) tongue tie (screen only), (6) non-nutritive suck, (7) bottle-feeding, (8) overall feeding skills and (9) recommendations. Results of the T-SP and IP-SP assessments were compared using agreement statistics. Parents reported perceptions of telepractice pre and post session, and also rated post-session satisfaction. The telepractice SP completed a satisfaction questionnaire post-appointment. The majority of assessment components (45/53, 85%) met the agreement criteria (≥80% exact agreement). Difficulties were noted for the assessment of palate integrity, gagging during non-nutritive suck assessment, and 6 components of the tongue tie screen. Parent and clinician satisfaction was high; SPs reported that they would offer telepractice services to 93% of families again in the future. Overall, the results demonstrated that most components of a bottle-feeding assessment could be reliably completed via synchronous telepractice in family homes. However, further research is required to improve the reliability of some intra-oral assessment components.

## 1. Introduction

Timely access to pediatric feeding assessment and treatment is critical for children with pediatric feeding disorders (PFDs) due to their potential negative impact on children and their families [1,2,3]. Whilst the COVID-19 pandemic has significantly impacted service access due to social distancing requirements [4], there is also a range of other factors that have historically negatively impacted access to feeding services. These include distance and travel, the speech pathologist’s skill and confidence, family commitments, and challenges to travelling with children with physical and/or medical needs [5,6,7,8,9,10,11]. Additionally, many traditional pediatric feeding services are provided within a clinical setting (i.e., not within the child’s usual feeding environment) and families have reported that their child’s feeding performance can differ between the home and clinical environment [9], suggesting that assessments conducted in a clinical environment may be less accurate than assessments performed in the home.

Telepractice has the potential to overcome some of these accessibility difficulties and could enable clinic-based services to provide appointments within the child’s home. However, clinicians have cited concerns regarding the safety and efficacy of this model of care as a barrier to uptake [12]. Within the adult population, clinical swallowing examinations (CSE) completed via telepractice are confirmed to be valid and reliable [13,14,15,16]. However, there are key differences between adult and pediatric swallowing assessments that make transferability of these findings limited.

To date, there are only a few published studies investigating the reliability of pediatric feeding assessments conducted via telepractice [17,18,19]. In 2012, Rojjansrirat et al. published their research investigating the feasibility and reliability of breastfeeding assessments conducted using the LATCH with 10 infant-mother dyads via telepractice [20]. Overall, they identified acceptable levels of agreement (>80% percentage exact agreement [PEA]) for 2/5 items during the first appointment, and 4/5 items during the second appointment. Whilst this telepractice model showed potential, the assessments were conducted with children without PFDs and did not comprehensively assess all of the elements typically included within a speech pathology infant feeding assessment (e.g., oral motor assessment, swallow safety).

More recently, Kantarcigil et al. [17] and Raatz et al. [18] have demonstrated the reliability of telepractice feeding assessments conducted with children with PFDs. Whilst Kantarcigil et al. [17] demonstrated substantial to excellent levels of agreement on the majority of items on the Dysphagia Disorders Survey and the Dysphagia Management Staging Scale [21], their study used an asynchronous design and evaluated the feeding skills of 19 children (aged 5–17 years) with cerebral palsy. More recently, Raatz et al. [18] used a synchronous telepractice model to investigate the feeding skills of 40 children (aged 4 months–7 years) with PFDs referred for assessment of their eating and/or cup drinking skills. They identified that the telepractice feeding assessments were reliable and acceptable to both clinicians and parents, with all assessment components except intraoral examination meeting agreement criteria (>80% PEA or 0.6 kappa) [18]. Whilst these findings are positive, and the assessment included some components commonly assessed during bottle feeding, the study focused solely on the investigation of solid feeding and/or cup drinking. Hence, the applicability of the findings to the assessment of bottle-feeding infants is limited.

To date, no known published research has investigated the reliability of bottle-feeding assessments conducted via telepractice. Whilst this study’s authors have developed and proposed a model for conducting synchronous bottle-feeding assessments via telepractice [22], pilot testing was only undertaken with one typically developing infant and further research is required to evaluate the model with infants with PFDs. Consequently, this study aimed to investigate whether acceptable levels of inter-rater reliability (PEA ≥ 80%; Kappa > 0.6) [18] could be achieved conducting bottle-feeding assessments via synchronous telepractice compared to traditional in-person methods using the system architecture described by Raatz et al. [22] Secondary aims were to investigate parent and clinician satisfaction associated with the telepractice appointments.

## 2. Materials and Methods

This study was conducted as part of a larger study examining the reliability of telepractice feeding assessments and the costs associated with a telepractice model of care. The results of the other studies are reported elsewhere [18,23]. Within this specific study, infants with bottle-feeding difficulties (including dysphagia) were recruited from an outpatient feeding service at the Queensland Children’s Hospital in Brisbane (Australia). This tertiary-level service provides statewide specialist feeding support for infants and children from 0–18 years with PFDs secondary to complex medical conditions affecting one or more body systems (e.g., cardiorespiratory, gastrointestinal, neurological). All bottle-feeding infants referred over a 6-month period who were eligible for this study were approached to participate. The research was conducted with ethical clearance from the Human Research Ethics Committees of both Children’s Health Queensland (HREC/17/QRCH/276) and The University of Queensland (2018001338).

### 2.1. Eligibility Criteria

Similar to Raatz et al. [18], to meet inclusionary criteria for this study, infants had to (1) be referred to speech pathology for assessment of bottle-feeding (i.e., not breastfeeding or solids), (2) reside within 40 km of the Queensland Children’s Hospital, (3) have internet access within their home, and (4) have a parent able to engage in the appointment without use of an interpreter. The distance criteria of 40 km was specified as the in-person SP (IP-SP) needed to travel to the family’s home for data collection. The language criteria was specified as the parent needed to respond to the direction of the telepractice SP (T-SP). There were no set minimum technology requirements as a loan device was available if required. Children were excluded from the study if they were in out-of-home care and/or environments deemed not suitable for a home visit (as identified on the health services ‘Home Visit Risk Assessment Plan’).

### 2.2. Evaluation Methodology

Evaluation methodology used in the study was consistent with that described in Raatz et al. [18] and previous adult CSE telepractice validation studies [16,24]. In summary, each child’s feeding was simultaneously assessed by two SPs; one SP who led the appointment via telepractice (the T-SP) and a second SP who was located at the child’s home (the IP-SP). Each SP assessed the infant’s feeding simultaneously, but independently, with both SPs instructed not to verbalize their clinical decisions to minimize potential bias [15,16,18,25]. The assessment was directed by the T-SP with the IP-SP observing. However, the IP-SP could touch the infant if required (e.g., to assess non-nutritive suck). This methodology enabled a comparison between the two SP assessment findings. For safety, the IP-SP could intervene during the appointment if there were clinical concerns that were not identified by the T-SP (e.g., inappropriate to continue with oral feeding trial).

Three SPs with pediatric feeding experience, but limited prior telepractice experience, participated in the study interchangeably. The SPs involved in each appointment were randomly assigned to the T-SP or IP-SP role for the assessment [18]. Given that multiple appointments were usually conducted within a day, SPs were randomized to their role (i.e., IP-SP or T-SP) for the day to minimize travel burden [18]. SP 1 (first author) participated in all appointments, while SP 2 and SP 3 alternated. Across the 30 assessments, SP 1 was the T-SP for 23% of appointments (*n* = 7), SP 2 for 60% (*n* = 18) and SP 3 for 17% (*n* = 5). All 3 SPs were trained to use the assessment form before the study commenced. Inter-rater reliability training was achieved by the SPs completing the assessment form simultaneously during in-person feeding assessments until at least 80% agreement was achieved on all components across 5 unique assessment sessions.

### 2.3. The Telepractice System

Telepractice appointments were conducted using the health service’s secure web-based videoconferencing portal. This portal was freely available to participants, and both families and SPs had access to free technical support via phone if required (e.g., difficulties with appointment connection). The portal used dual image layout and a bandwidth of >0.4 Mbps. Consistent with Raatz et al. [18], the T-SP linked into the appointment using a desktop computer, Logitech 1080p HD Pro webcam and Jabra SPEAK 410 MS speaker, whilst participants used their own personal equipment (i.e., consumer-grade laptop, tablet, or smart phone). If the family required a loan device, then the IP-SP loaned them a smart phone for the session. The IP-SP also provided families with a loan Joby Gorillapod Tripod (https://joby.com/au-en/gorillapod-1k-jb01503-config/ (accessed on 21 October 2020)) to help position smaller devices (phones or tablets) if required [18].

### 2.4. The Assessment Form

A purpose-built assessment form (attached in Supplementary Materials) was developed as there was no available standardized assessment that met the study needs [26,27,28,29]. The assessment form was developed to incorporate assessment measures/parameters typically completed during an in-person infant feeding assessment [30,31,32,33,34,35,36]. The developed form incorporated 53 assessment items across nine areas. Individual sections included (1) developmental screen, (2) assessment of state, color and respiration, (3) oral motor assessment, (4), infant oral reflex exam, (5) tongue tie screen, (6) non-nutritive suck assessment, (7) assessment of bottle-feeding, (8) assessment of overall feeding skills and (9) recommendations. As per Raatz et al. [18], the form incorporated forced choice ratings of overall assessment components (i.e., within normal limits vs. different from expectations), and the SP then marked the observed difference/s from a set list [18]. Children’s overall feeding skills were rated using both the Eating and Drinking Classification Scale (EDACS) and the Functional Oral Intake Scale-Suckle Feeds and Transitional Feeds (FOIS-SFTF) [30,37]. Both tools were incorporated due to their different focus areas. As the study investigated bottle feeding, only the 6 relevant FOIS-SFTF scores were utilized (i.e., 4 and 4.5 were collapsed). The developed assessment form was reviewed for content and clinical applicability by a group of 10 SPs with pediatric feeding experience (ranging from 4–20 years). Following this, the form was piloted in four in-person feeding assessments to confirm relevance and useability.

### 2.5. Telepractice Sessions

**Figure 1:**
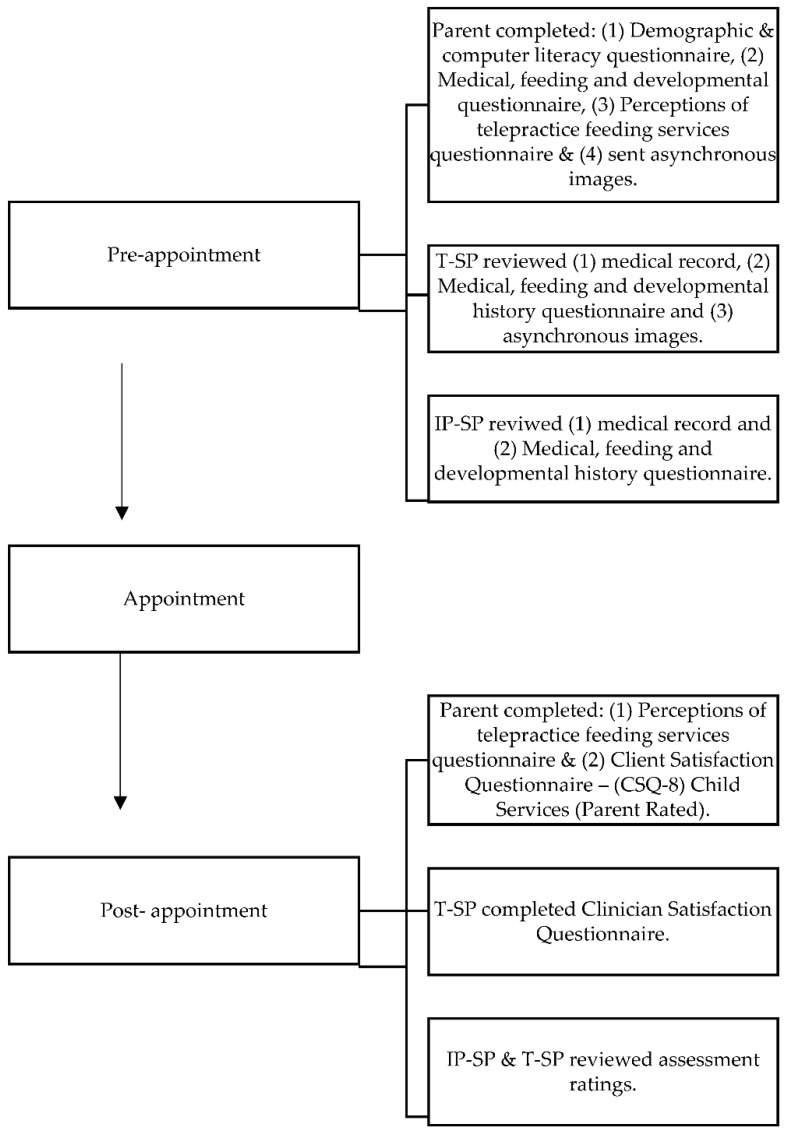
Overview of telepractice appointment procedures.

The telepractice sessions were conducted using the system architecture and methods described by Raatz et al. [18,22]. A summary of these processes is outlined in Figure 1 and described below.

#### 2.5.1. Pre-Appointment

Prior to their telepractice appointment, parents were emailed two fact sheets; one provided them with information about preparing for the appointment (e.g., optimizing lighting, what to have prepared) and the other provided information on how to capture asynchronous images to send to the T-SP [18,22]. Parents were asked to send photos of (1) their child’s usual position for feeding, (2) the inside of their child’s mouth and (3) their child’s tongue when elevated. Before the session, parents were asked to complete three questionnaires, including (1) a medical, feeding and developmental questionnaire to gather case history information, (2) a demographic and computer literacy questionnaire, and (3) the perceptions of telepractice feeding questionnaire (pre-session format) [18]. The Demographic and computer literacy questionnaire (adapted from [38]) is an 11-item questionnaire that collected information about parent’s demographics, technology use and confidence, and use of technology for health-related activities. The Perceptions of Telepractice Feeding Services (adapted from [24,25]) is a 16-item Likert-rated questionnaire investigating parent’s perceptions of telepractice for assessment of their child’s feeding skills. This questionnaire was used pre and post session to enable an exploration of changes in parent perception before and after the session through modifications to the question wording, e.g., ‘I will be (was) comfortable using technology for my child’s appointment’ [18].

#### 2.5.2. Telepractice Appointment

One-hour telepractice appointments were scheduled within weekly clinics. Prior to the appointment, the IP-SP and T-SP independently reviewed the child’s electronic medical record and the medical, feeding and developmental history questionnaire. The T-SP also reviewed the asynchronous images. During the appointment, the T-SP was in a clinical room at the Queensland Children’s Hospital and the IP-SP was located at the family’s home. Camera positions for the telepractice appointment were consistent with the system architecture described by Raatz et al. [22] and three main camera angles were used: (1) a wide-angle front on view for general observations, (2) a close-up view for tasks such as the infant oral reflex exam and (3) a 45-degree angle for observation of bottle feeding.

The T-SP confirmed emergency and disconnection procedures at the start of each appointment and asked the parent what device they were using to link into the appointment [18]. Infants were then observed bottle-feeding, with some children observed on more than one equipment/positioning set up (e.g., medium flow and slow flow teat). Both the T-SP and IP-SP simultaneously scored the child’s feeding skills using the previously described assessment form. The T-SP could request verbal clarification from the parent as necessary (e.g., “It sounds like they have a cold. Is that what they sound like to you?”). Assessment results were then compared at the end of the day with the SPs discussing potential reasons for rating discrepancies [18].

#### 2.5.3. Post-Session Feedback

After their appointment parents completed the Client Satisfaction Questionnaire—(CSQ-8) Child Services (Parent Rated) [39,40] and the Perceptions of Telepractice Feeding Services questionnaire (post session format). The CSQ-8 is a validated 8-item tool assessing parent satisfaction with healthcare services, with responses rated on a 4-point Likert scale [39]. Parents completed a hard copy of the CSQ-8 and could choose to complete the Perceptions of Telepractice Feeding Services questionnaire either in hard copy or electronically. Hard copy questionnaires could be returned to the IP-SP post session or via a reply paid envelope (as per parent preference).

Post session, the T-SP also completed the Clinician Satisfaction Questionnaire. This is a 19-item non-standardized questionnaire adapted by Raatz et al. [18] from prior telepractice studies to be applicable to the pediatric context [15,16,24,41,42]. The questionnaire explored assessment ability, video and audio quality, perceptions of the effectiveness of using telepractice to evaluate bottle-feeding and whether they would offer telepractice appointments for that child again in the future. Questions were rated on a 5-point Likert Scale, with one free-text response option.

### 2.6. Data Analysis

Descriptive analysis was used to analyze demographics, session data, clinician satisfaction ratings and CSQ-8 scores. Ratings from the telepractice and in-person conditions were analyzed using a non-inferiority method-comparison design [43,44]. Agreement between raters are reported as percentages, with percentage exact agreement (PEA) set at≥ 80% and >0.6 Kappa as per prior telepractice research [15,16,25]. Scores on the Perceptions of Telepractice Feeding Services Questionnaire were collated into 3 groups (disagree/unsure/agree) and are reported descriptively. Data from the pre- and post-session format of this questionnaire were directly compared to determine any changes in perceptions after the telepractice appointment and were analyzed using Chi-square or Fisher’s exact test with significance set at *p* < 0.05.

## 3. Results

### 3.1. Participants

Overall, 33 infants were initially consented to participate in the study. Two later became ineligible to participate (1 moved out of catchment, 1 became not suitable for home visit due to change in circumstances) and significant technical difficulties prevented 1 appointment from being completed. Hence, data were available for 30 participants for analysis.

#### 3.1.1. Child Characteristics

Children were aged 1 month–2 years, with detailed medical and feeding background information outlined in Table 1. There were slightly more females (*n* = 17) than males (*n* = 13). Eleven infants had a feeding tube (*n* = 8 nasogastric tubes and *n* = 3 gastrostomy tubes) and 33% (*n* = 11) were on home oxygen. Thirteen children (43%) were seen for initial assessments and 17 (57%) attended follow-up assessments. Of the 17 children attending for follow-up appointments, 2 were not known to either assessing SP, 7 were known to T-SP only, 4 were known to IP-SP only, and 4 were known to both SPs. As per Raatz et al. [18], to minimize any potential bias, the SPs were instructed to assess the infant based on the behaviors observed within the telepractice session only (i.e., no prior knowledge of the child).

#### 3.1.2. Parent Demographic and Technology Information

Demographic information was provided for 29 families (one parent did not return questionnaire) and is detailed in Table 2. All parents (100%) had a partner and most (76%) were aged between 26–44 years. The majority of parents (76%) reported that they felt confident using an electronic device for health-related activities, and no parents reported that they required support to use the device/s.

### 3.2. Telepractice Sessions

All telepractice sessions were completed without the need for the IP-SP to intervene. Only 9 parents (30%) sent photos of their child’s oral cavity prior to the appointment as requested. Information was not collected regarding reason/s for not completing this request.

#### 3.2.1. Primary Aim: Inter-Rater Reliability

High levels of agreement (PEA ≥ 80% and Kappa > 0.6) were achieved for most assessment elements (41/53) as outlined in Table 3. There were four other assessment elements (rhythmicity, oral phase, nasal congestion and implementation of feeding skills) where the Kappa value was below the set criteria but PEA was >80% so these were considered to meet agreement criteria, leading to 85% of items considered to meet reliability criteria (45/53).

Eight assessment items (over 3 components-infant oral reflex exam (*n* = 1), tongue-tie screen (*n* = 6) and non-nutritive suck assessment (*n* = 1)) did not meet agreement criteria (Table 3). Regarding the assessment of palate integrity, it is important to note that this item was considered difficult to assess in both the in-person and telepractice conditions and was unable to be completed by either SP in 14 of the appointments (47%). However, for the children where this assessment component was able to be assessed, the provision of asynchronous images pre-appointment was beneficial and improved agreement (71% PEA for palate integrity ratings when images were provided vs. 11% PEA without images, *p* = 0.01).

Three other assessment tasks (infant oral reflex exam, tongue-tie screen and non-nutritive suck assessment) were also not completed for every participant, either due to age (i.e., not appropriate for their age) or due to child distress. The infant oral reflex exam was only able to be completed by both SPs during 17 appointments (57%); this parameter was not attempted during 12 appointments (40%) and was able to be completed by the IP-SP but not the T-SP on one occasion (3%). For the appointments where both SPs were unable to complete the assessment, all assessment elements except gag were able to be reliably completed via telepractice.

The tongue-tie screen was only able to be completed by both SPs for 13 participants (43%). This assessment component was not attempted by either SP in 10 appointments (33%), was unable to be completed by T-SP but completed by IP SP during five appointments (17%) and was able to be completed by T-SP but not the IP-SP in two appointments (7%). The majority of components of the tongue tie screen (*n* = 6) did not meet the specified agreement criteria. The overall assessment rating (i.e., within normal limits vs. concerns) demonstrated high reliability; however, assessment of individual components (e.g., frenulum thickness) did not. Completion of the tongue tie screen had slightly higher levels of agreement (71% vs. 54%) when asynchronous images were available; however, this difference was not statistically significant (*p* = 0.47).

The non-nutritive suck assessment was completed by both SPs during 17 appointments (57%) and was not attempted during 10 appointments (33%) and was unable to be completed by the T-SP on three occasions (10%). Where completed, all assessment components met the specified PEA criteria.

#### 3.2.2. Secondary Aims: Clinician Satisfaction

Clinicians were reportedly highly satisfied with the telepractice appointments and almost all (93%) reported they would re-offer telepractice services again (Table 4). In free-text comments, clinicians reported improved assessment ability within the infant’s natural, home environment as a perceived benefit of the telepractice appointment. For example, one clinician stated, “[it] was useful to see positioning at home (lying flat on bean bag) that wouldn’t have been possible in clinic”. There were some difficulties noted however, with reduced sound/image quality reported as reasons why clinicians would not re-offer telepractice services to two children again in the future. Two SPs also provided free-text comments about the difficulty of completing the tongue-tie screen via telepractice due to their lack of an ability to be ‘hands on’.

#### 3.2.3. Secondary Aims: Parent Perceptions (CSQ-8 and Perceptions of Telepractice Feeding Services)

Complete questionnaire data was obtained from 23 parents (1 did not complete pre-appointment questionnaires and 6 did not complete post-appointment questionnaires). Additionally, two parents did not complete the final four questions on the post-appointment Perceptions of Telepractice Feeding Services questionnaire. It is anticipated that this was accidental, as these four questions were on the back of a double-sided form. Another two parents did not provide an answer for the question “my child was able to establish rapport with the telepractice speech pathologist”, noting that their child was too young to provide an answer to this.

Overall, parents reported high satisfaction with the telehealth session (Table 5). They also reported positive perceptions about telepractice both before and after the appointment (Table 6). A direct comparison of the pre and post session perceptions data revealed little change for most items (Table 6); however, a significant (*p* = 0.016) shift was noted post session regarding children developing a rapport with the online clinician and perceptions of telepractice improving access to healthcare (*p* = 0.004). Seven parents provided free-text comments about their child’s telepractice appointment. Four comments provided positive feedback about the telepractice appointment (e.g., “I really enjoyed the help and support it is very clear quality. I would highly recommend this new program! It’s amazing!”), two reported their preference for both telepractice and in-person appointments for their child’s future care, and one reported that whilst they saw the benefits of the telepractice appointment for families who did not have easy access to in-person services, their personal preference remained to access in-person care.

#### 3.2.4. Device Use and Technical Difficulties

The majority of parents (*n* = 14) linked into their child’s appointment using a smart phone (laptop *n* = 10, tablet *n* = 4). One appointment had to be rescheduled due to audio issues that were unable to be resolved; however, the appointment was successfully completed during the rescheduled telepractice appointment. The IP-SP provided a loan device to parents during four appointments. The loan device was required by two parents because their device ran out of battery, and for the other two parents due to video/audio issues that were unable to be resolved with their own device. The T-SP reported technical difficulties during eight other appointments incorporating (1) session dropouts that were resolved during three appointments and (2) variable audio/video quality during five appointments. There were four comments by SPs regarding auditory quality; two reported their satisfaction with the high sound quality experienced during the telepractice appointment, one discussed that although they were able to hear coughing and stridor during feeding, these were sometimes difficult to distinguish, and one described difficulties in detecting nasal congestion.

## 4. Discussion

This study aimed to determine whether acceptable levels of inter-rater reliability could be achieved conducting bottle-feeding assessments via telepractice compared to the traditional in-person model. Overall, study results identified that the majority of elements of a clinical bottle-feeding assessment could be reliably completed via telepractice and that the telepractice model was acceptable to both parents and SPs. These findings are consistent with previous research in other areas of feeding/swallowing [13,14,15,16,17,18,19].

However, results also highlighted that completion of some assessment components were difficult to reliably complete via telepractice. Future research should investigate potential solutions to gather this information more reliably via telepractice, including the capture of asynchronous information. The specific assessment tasks that were noted to be difficult to complete via telepractice were traditionally ‘hands on’ tasks, where the SP routinely touches the child’s face and mouth during an in-person consultation. The difficulties noted with completion of the assessment of palate integrity are consistent with previous pediatric research [18]. This research also identified that asynchronous images improved the telepractice assessment ability, but compliance from participants was low, which prompts the need for research into why this was challenging for families. SPs should be cognizant of the limits of certain components of infant-feeding assessments conducted via telepractice, and should carefully consider whether their client is appropriate for an initial assessment via telepractice. However, given that these tasks are typically only completed within an initial assessment (i.e., not required during subsequent appointments), these limitations would typically not apply to follow-up appointments conducted via telepractice. Additionally, in the context of growing evidence supporting the feasibility and potential of tele-supervision [45], clinicians could use telepractice to provide shared appointments to enable observation opportunities, mentoring, feedback and/or specialist support to develop greater skill and confidence in the completion of the clinical tasks required during an initial feeding assessment via telepractice.

Similar to previous research, clinicians cited the ability to assess the child’s feeding in their home environment as a perceived benefit of the telepractice assessment model [12,18]. Given the importance of evaluating the impact of the environment and environmental factors during feeding [46], this finding is particularly pertinent for providers who are unable to provide home-visiting services. Indeed, telepractice may afford providers with the opportunity to better understand the child’s typical feeding behaviors [2,10] and/or to obtain an assessment that better represents the infants’ usual feeding in their home environment [9,47]. Telepractice may also help to reduce the burden that some families can experience in accessing in-person services [2,9,10,23]. Parent feedback highlighted the importance of offering a choice to families for service access options, with many parents wanting to be offered a combination of telepractice and in-person appointments. Additionally, as novel service methods, such as neonatal tele-homecare [48,49], become more common-place, the ability to offer telepractice bottle-feeding appointments would ensure that families of children at risk of difficulties could readily access speech pathology feeding support as they transition home.

Finally, technical difficulties and/or concerns regarding the impact of potential technical difficulties have previously been highlighted as barriers to telepractice service provision [12,50]. Within this study, a number of technical difficulties were experienced. Of note, two families required the use of a loan device because their device ran out of battery. This highlights the importance of ensuring that families have adequately charged their device/s prior to their appointment and/or ensuring they are connected to a power source throughout the appointment. Additionally, a few families experienced initial difficulties logging into the appointment, and two had difficulties establishing video/audio. These difficulties highlight the importance of pre-appointment preparation (e.g., conducting test calls with families) and/or readily available technical support.

### Limitations

This study has several limitations, many of which were unavoidable, but do not invalidate or undermine the value of this research. Due to similarities in study design, a number of these limitations are similar to those reported by Raatz et al. [18]. Firstly, there are limitations to the synchronous (real-time) study design [44]. Although this design was used to reduce potential variances in child performance between two time points, the presence of an SP in the home may have increased parent confidence in the telepractice appointment. It is also acknowledged that a clinical feeding assessment is a subjective evaluation and consequently there is potential for variability due to numerous factors. Although inter-rater reliability training was completed with the SPs prior to data collection to try to reduce evaluation subjectivity, this limitation still needs to be acknowledged.

There is also a potential level of bias in satisfaction and perceptions data, recognizing that participants (SPs and parents) opted to be involved in the study; there may have been unconscious bias and/or greater acceptance of telepractice services. Indeed, ratings on the perceptions questionnaire conducted prior to the assessment identified that the majority of families were already reporting neutral or positive perceptions of telepractice. It is also acknowledged that parents who required an interpreter were excluded from participation in this study, and families from culturally and linguistically diverse backgrounds and/or those who require interpreters, may have different experiences or perceptions of telepractice. Another potential source of bias is that the majority of children within this study were known to one or both SPs. Although SPs were directed to only assess the infant’s behaviors during the assessment appointment only, this potential level of bias needs to be acknowledged. Finally, all participants in this study were recruited from one service, which has implications for the generalizability of these results across different service contexts and populations. Future research is needed to examine the reliability of telepractice bottle feeding assessments with a cohort of infants with more severe PFDs, to examine the perceptions of telepractice of families from diverse cultural and linguistic backgrounds and across different service contexts, and to continue to develop the current evidence base supporting telepractice use for bottle-feeding assessments in clinical practice.

## 5. Conclusions

This is the first known, published study investigating telepractice to provide bottle-feeding assessments. Results support that the majority of components of a bottle-feeding assessment conducted via telepractice were feasible to conduct and the data was reliable when compared to an in-person assessment. The data also confirmed that conducting bottle-feeding assessments via telepractice was acceptable to clinicians and parents. Future research that examines ways to optimize the assessment of some items, including those traditionally ‘hands on’ assessment components, will help to further develop the potential of telepractice to support the remote assessment of bottle-feeding infants.

## Figures and Tables

**Table 1:** Infant’s medical and feeding background (*n* = 30).

Characteristics	*n* (%)
Age (Corrected)	
1–3 months	9 (30)
4–6 months	9 (30)
7–11 months	5 (17)
1–2 years	7 (23)
Medical diagnose/s	
History of prematurity	17 (57)
Neurological	5 (17)
Respiratory	19 (63)
Gastroenterology	7 (23)
Allergy/Immunology	5 (17)
Cardiac	4 (13)
Developmental	2 (7)
Musculoskeletal	3 (10)
Genetic	4 (13)
Renal	3 (10)
Cleft/Craniofacial	1 (3)
Structural/ENT (e.g., laryngeal cleft)	6 (20)
Number of body systems impaired	
0	1 (3)
1	11 (37)
2	10 (33)
3	7 (23)
7	1 (3)
FOIS-SFTF Rating (1 = most severe impairment) *n* = 29	
1 (No oral intake)	0 (0)
2 (Tube dependent with minimal oral intake)	1 (3)
3 (Tube dependent with consistent oral intake)	10 (34)
4 (Total oral diet requiring special preparation)	2 (7)
5 (Total oral diet requiring compensations)	9 (31)
6 (Total oral intake with no restriction relative to peers)	7 (24)
EDACS Rating (5 = most severe impairment) *n* = 29	
1 (Eats/drinks safely and efficiently)	7 (24)
2 (Eats/drinks safely, but with limitations to efficiency)	14 (48)
3 (Eats/drinks with some limitations to safety)	6 (21)
4 (Eats and drinks with significant limitations to safety)	2 (7)
5 (Unable to eat or drink safely)	0 (0)

FOIS-SFTF = Functional Oral Intake Scale-Suckle Feeds and Transitional Feeds [30]. EDACS = Eating and Drinking Classification Scale [37].

**Table 2:** Parent demographics and technology experience/confidence (*n* = 29) [18].

Characteristics	*n* (%)
Age	
18–25 years	4 (14)
26–34 years	15 (52)
35–44 years	7 (24)
Not specified	3 (10)
Highest level of education	
Grade 10 or below	4 (14)
Grade 12	3 (10)
Certificate or Diploma	13 (45)
Bachelor’s Degree	9 (31)
Usual device/s	
Desktop computer	11 (38)
Laptop computer	20 (69)
Tablet	15 (52)
Smart phone	29 (100)
Confidence using device/s for everyday activities	
Very confident	17 (59)
Somewhat confident	11 (38)
Not confident	0 (0)
No response	1 (3)
Previously used device/s for online health related activities	
Yes	12 (41)
No	17 (59)
Confidence using device/s for online health related activities	
Very confident	8 (28)
Somewhat confident	14 (48)
Unsure	5 (17)
Somewhat not confident	1 (3)
No response	1 (3)

**Table 3:** Inter-rater reliability for clinical feeding assessment across in-person and telepractice conditions (*n* = 30).

Assessment Component	Percentage Exact Agreement (PEA)	Kappa Coefficient
Developmental screen	98	0.84
State		
Before feed	96	0.77
During feed	93	–
After feed	96	0.82
Color		
Before feed	100	1.00
During feed	100	–
After feed	100	1.00
Pre-feeding respiration	99	–
Oral sensorimotor assessment		
Total agreement	89	–
Face	99	–
Lips	99	0.65
Tongue	93	–
Fat pads	93	–
Jaw	99	0.84
Saliva control	90	–
Palate	67 ^a^	0.31 ^b^
Cry	83	0.62
Infant Oral Reflex Exam (*n* = 17)		
Rooting reflex	94	0.85
Tongue protrusion reflex	82	0.69
Transverse tongue reflex	100	1.00
Phasic bit	94	0.77
Gag	76 ^a^	0.53 ^b^
Tongue Tie Screen (*n* = 13)		
Overall rating (within normal limits vs. concerns)	92	0.63
Tongue posture during crying	54 ^a^	0.03 ^b^
Shape of elevated tongue	100	1.00
Tongue lateralization	62 ^a^	0.24 ^b^
Lingual frenulum	54 ^a^	0.23 ^b^
Frenulum thickness	61 ^a^	0.27 ^b^
Frenulum attachment to tongue	54 ^a^	0.32 ^b^
Extension of tongue	69 ^a^	0.29 ^b^
Non-nutritive Suck assessment (*n* = 17)		
Total agreement	90	–
Response to stimulus	94	0.77
Positive pressure generation	100	–
Negative pressure generation	94	0.88
Rhythmicity	88	0.43 ^b^
Jaw excursion	94	0.64
Tongue cupping (*n* = 8)	88	0.75
Bottle feeding (*n* = 42)		
Oral phase	92	0.52 ^b^
Suck-swallow	98	0.93
Physiological stability	98	0.78
Disengagement cues	91	1.00
Feeder response to infant cues	100	1.00
Respiratory changes	95	0.83
Indicators of penetration +/- aspiration	97	0.68
Nasal congestion/regurgitation	98	0.48 ^b^
Overall assessment		
Within normal limits vs impaired	100	1.00
FOIS-SFTF rating	100	1.00
EDACS rating	100	1.00
Recommendations		
Fluid level	100	1.00
Equipment change	100	1.00
Positioning change	97	0.93
Implementation of feeding strategies	90	0.53 ^b^
Session outcome (discharge vs. urgent review vs. non-urgent review)	100	1.00

^a^ <80% criteria. ^b^ <0.6 kappa. FOIS-SFTF = Functional Oral Intake Scale-Suckle Feeds and Transitional Feeds [30]. EDACS = Eating and Drinking Classification Scale [37].

**Table 4:** Clinician satisfaction with telepractice assessments (*n* = 29) [18].

Parameter	1 Strongly Disagree	2	3 Neutral	4	5 Strongly Agree	Median
Effective service delivery method for appointment reason	0	1 (3%)	2 (7%)	15 (52%)	11 (38%)	4
Established rapport with child	0	1 (3%)	14 (48%)	12 (41%)	2 (7%)	3
Established rapport with parent/carer	0	1 (3%)	1 (3%)	16 (55%)	11 (38%)	4
Managed child’s behavior	0	0	12 (41%)	15 (52%)	2 (7%)	4
Able to assess feeding skills	0	1 (3%)	3 (10%)	14 (48%)	11 (38%)	4
Able to assess swallow safety	0	1 (3%)	3 (10%)	20 (69%)	5 (17%)	4
Able to assess oral sensorimotor skills	0	2 (7%)	4 (14%)	16 (55%)	7 (24%)	4
Assessing child in the home environment improved clinical decision-making	0	0	4 (14%)	11 (38%)	14 (48%)	4
Information gathered via in-home telepractice that would not have been gathered during in-clinic appointment	0	2 (7%)	3 (10%)	12 (41%)	12 (41%)	4
Video quality adequate for general session	0	2 (7%)	2 (7%)	15 (52%)	10 (34%)	4
Video quality adequate for diagnostic purposes	0	3 (10%)	7 (24%)	15 (52%)	4 (14%)	4
Audio quality adequate for general session	1 (3%)	1 (3%)	0	15 (52%)	12 (41%)	4
Audio quality adequate for diagnostic purposes	1 (3%)	2 (7%)	7 (24%)	16 (55%)	3 (10%)	4
Would use telepractice to provide feeding services again for this child	0	2 (7%)	0	17 (59%)	10 (34%)	4

**Table 5:** Post-appointment parent satisfaction with telepractice assessment using the Client Satisfaction Questionnaire-8 (*n* = 23) [39,40].

Parameter	1 = Low	2	3	4 = High	Mean
Quality of services provided to child	0	0	3	20	3.9
Received the kind of service wanted	0	0	7	16	3.7
The service met child’s needs	0	1	8	14	3.6
Would recommend service to friend’s child in need of similar help	0	0	8	15	3.7
Satisfaction with amount of help child received	0	0	7	16	3.7
Services have helped child to deal more effectively with problems	0	0	10	13	3.6
Satisfaction with services child received	0	0	4	19	3.8
Would come back	0	0	5	18	3.8

The item content of the CSQ-8 is abstracted here to enhance information value of this table. The CSQ-8 is a copyright instrument and was used in this study with express written permission of the copyright holder. For additional information please consult csqscales.com, accessed on 25 October 2021.

**Table 6:** Parents’ pre-post perceptions of telepractice assessments using the Telepractice for Feeding Questionnaire (*n* = 23) [18].

Item	Pre-Assessment			Post-Assessment				Chi-Square/ Fisher’s Exact
	Disagree	Unsure	Agree	Disagree	Unsure	Agree	No Response (post)	
I *feel* (felt) comfortable having my child’s feeding and/or swallowing skills assessed via telehealth	0	1 (4%)	22 (96%)	0	1 (4%)	22 (96%)	0	0.758
I *am* (was) comfortable using technology for my child’s appointment	0	2 (9%)	21 (91%)	0	0	23 (100%)	0	–
The telepractice feeding assessment *will save* (saved) me time (e.g., time spent travelling to appointment)	1 (4%)	1 (4%)	21 (91%)	0	0	23 (100%)	0	–
The telepractice feeding assessment *will save* (saved) me money (e.g., bus fare, parking)	0	0	23 (100%)	0	0	23 (100%)	0	–
It *will be* (was) easy to set up for the telepractice appointment	0	7 (30%)	16 (70%)	1 (4%)	1 (4%)	21 (91%)	0	0.283
I feel that the online feeding assessment *will be* (was) equal to having a face to face feeding assessment	1 (4%)	10 (43%)	12 (52%)	1 (4%)	3 (13%)	19 (83%)	0	0.129
I *will have* (had) opportunities to clarify any questions I had during the online assessment	0	3 (13%)	20 (87%)	0	1 (4%)	22 (96%)	0	0.875
I *will be* (was) able to manage my child’s behavior during the telepractice assessment	1 (4%)	6 (26%)	16 (70%)	0	2 (9%)	21 (91%)	0	0.776
I *feel* (felt) the telepractice feeding assessment will accurately represent(ed) my child’s usual feeding and swallowing skills	0	8 (35%)	15 (65%)	1 (4%)	0	22 (96%)	0	0.130
Having the telepractice appointment in our home *will improve* (improved) the speech pathologist’s understanding of my child’s feeding skills and behavior	0	3 (13%)	20 (87%)	1 (4%)	2 (9%)	20 (87%)	0	0.236
I *will be* (was) able to establish rapport with the telepractice speech pathologist	0	1 (4%)	22 (96%)	0	1 (4%)	22 (96%)	0	0.958
My child *will be* (was) able to establish rapport with the telepractice speech pathologist	0	8 (35%)	15 (65%)	2 (9%)	3 (13%)	14 (61%)	4 (17%)	0.016 *
I feel that a telepractice feeding assessment can replace a face-to-face feeding assessment	1 (4%)	9 (39%)	13 (57%)	1 (4%)	5 (22%)	15 (65%)	2 (9%)	0.07
I feel telepractice will improve easy access to healthcare	0	2 (9%)	21 (91%)	0	2 (9%)	19 (83%)	2 (9%)	0.004 *
I feel telepractice will be beneficial for other children with feeding difficulties	0	5 (22%)	18 (78%)	1 (4%)	2 (9%)	18 (78%)	2 (9%)	0.557

* *p* = <0.05. Pre-appointment questionnaire wording in italics, and post-appointment questionnaire wording in brackets.

## Data Availability

The dataset generated during this study is not publicly available to maintain the confidentiality of the participants and the relevant health services. Access to this data may be available through request to the authors and relevant human research and ethics committee.

## Supplementary Materials

The following are available online at https://www.mdpi.com/article/10.3390/children8110989/s1, S1: Bottle-feeding assessment form.
