# Supplementary material for: Evaluating the Use of Telepractice for Bottle-Feeding Assessments
Source: Children (Basel). 2021 Nov 1;8(11):989. doi: 10.3390/children8110989 (PMC8625576; doi:10.3390/children8110989)
Supplement: Supplementary file 1 [file children-08-00989-s001.zip › children-1397786-supplementary.pdf]

S1: Bottle-feeding Assessment form

**1. Introduction, safety procedures and disconnection procedures** Camera Position 1 – Wide angle view of parent and child	
☐	**1. Introduction:** Introduce yourself, the purpose of the session and clarify who is present
☐	***2.*** **Emergency procedures:** Verbally establish and clarify the emergency procedures:<br>☐ Provide the family with your contact number<br>☐ Clarify the family's best contact number<br>☐ Verbal agreement with plan in case of emergency e.g. "*In the case of a medical emergency, you are responsible for commencing first aid and for calling emergency services. I will stay on the line and can provide you with support as needed. Do you understand what to do in case of an emergency?"*
☐	**3. Disconnection procedures:** Verbally establish and clarify what to do in the event of a session disconnection e.g. - *"If the appointment cuts out and doesn't reconnect within 1 minute, try logging out of the appointment and re-connecting. If you are still unable to establish a connection please call me on the phone number I've given you"*

**2. Case history and review of sent images** [1]<br>Camera Position 1 – Wide angle view of parent and child	
☐	**1. Case History:** Review the case history information received prior to the telehealth session and clarify/discuss further information as needed.
☐	***2.*** **Discuss images:** Discuss the photos sent through prior to the appointment and clarify that the picture/s are representative of the child's usual feeding position. If the soft palate and uvula are not visible on the photo, consider asking the following question:<br>▪ Does the child ever have milk come out of their nose (nasal regurgitation)?<br>○ If the parent answers yes and you are unable to fully assess the integrity of the palate suggest local assessment (e.g. GP, Child Health Nurse, Speech Pathologist)

**3. Developmental screen** [1]<br>Camera position 2 – Full view of child's head and body with child positioned on flat surface (e.g. cot) or in supportive seating position (e.g. bouncer). The parent will need to have both hands free.
Observe the child playing with a toy for 1-2 minutes. Use this time to informally screen the child's overall development; note gross motor screen may be incomplete due to positioning of the child.<br><br>☐ WNL (No concerns)<br>☐ Unable to assess<br>☐ Concerns in the following area/s:<br>☐ Gross motor ☐ Fine motor ☐ Play ☐ Communication ☐ Other<br><br>Comments:

Raatz, M., Ward, E.C., Marshall, J., Burns, C.L.

**4. Infant state, color and respiration** [2]<br>Camera position 2. *Note- you will need to complete the 'during' and 'after' feeding section at the appropriate time during the assessment.*			
	**State**	**Color**	**Respiration**
**Before feed**	☐ Deep Sleep<br>☐ Light sleep<br>☐ Drowsy<br>☐ Quiet alert<br>☐ Active alert<br>☐ Alert agitated<br>☐ Crying	☐ Well perfused<br>☐ Pale<br>☐Dusky<br>☐ Mottled<br>☐ Cyanotic<br>☐ Flushed<br>☐ Grey	☐ Tracheal tug<br>☐ Recessions<br>☐ Stridor<br>☐ Apnoea<br>☐ Congested breathing<br>☐ Cough<br>☐ Wet airway noise
**During feed**	☐ Deep Sleep<br>☐ Light sleep<br>☐ Drowsy<br>☐ Quiet alert<br>☐ Active alert<br>☐ Alert agitated<br>☐ Crying	☐ Well perfused<br>☐ Pale<br>☐Dusky<br>☐ Mottled<br>☐ Cyanotic<br>☐ Flushed<br>☐ Grey	Mark on feeding observations
**After feed**	☐ Deep Sleep<br>☐ Light sleep<br>☐ Drowsy<br>☐ Quiet alert<br>☐ Active alert<br>☐ Alert agitated<br>☐ Crying	☐ Well perfused<br>☐ Pale<br>☐Dusky<br>☐ Mottled<br>☐ Cyanotic<br>☐ Flushed<br>☐ Grey	

**5. Oral motor assessment** [2, 3, 4]<br>Camera position 2 (full view of head/body) + asynchronous photos		
Assess the child's oral motor skills during play/at rest. If no differences are observed tick 'NAD'. If differences are observed, tick areas of difference/concern the side (left- L or right – R) if applicable.		
**Face**<br>☐ NAD<br>☐ Unable to assess	☐ Asymmetrical at rest L R<br>☐ Hyposensitive response to touch	☐ Asymmetrical in movement L R<br>☐ Hypersensitive response to touch
**Lips**<br>☐ NAD<br>☐ Unable to assess	☐ Asymmetrical at rest L R<br>☐ Other (e.g. cleft, scar)	☐ Asymmetrical in movement L R<br>☐ Reduced rate of movement
**Tongue**<br>☐ NAD<br>☐ Unable to assess	☐ Asymmetrical at rest L R<br>☐ Impaired protrusion<br>☐ Fasciculations	☐ Asymmetrical in movement L R<br>☐ Impaired lateralisation<br>☐ Reduced rate of movement
**Fat Pads**<br>☐ NAD	☐ Absent	
**Jaw**<br>☐ NAD<br>☐ Unable to assess	☐ Asymmetrical at rest L R<br>☐ Hanging open at rest<br>☐ Micrognathia/retrognathia	☐ Asymmetrical in movement L R<br>☐ Clenched
**Palate**<br>☐ NAD<br>☐ Unable to assess	☐ Soft palate cleft<br>☐ Requires further assessment (report of nasal regurgitation, unable to visualise palate fully)	☐ Hard and soft palate cleft
**Saliva control**<br>☐ NAD<br>☐ Unable to assess	☐ Thick/vicious saliva	☐ Unable to manage secretions
**Cry**<br>☐ NAD<br>☐ Unable to assess	☐ Weak	☐ Other

**6. Infant oral reflex exam** [4] ☐ Not attempted ☐ Unable to complete<br>Camera position 3 – Close up view of the child's mouth. The parent/carer will need to have both hands free.				
Instruct the parent/carer how to conduct the exam using the specific instructions below				
**Reflex**	**Diminishes**	**Parent instruction**	**Present**	**Not stimulated**
Rooting	4-6 months	"Gently touch the side of their mouth and see if they turn towards you"	☐	☐
Tongue protrusion	4-6 months	"Gently touch the tip of their tongue and see if they poke their tongue out"	☐	☐
Transverse tongue	4-6 months	"Gently touch the side of their tongue and see if their tongue moves towards your finger"	☐	☐
Phasic bite	9-12 months	"Gently rub your finger along their gums and see if they try to bite down"	☐	☐
Gag	Continues into adulthood	* Not to be directly assessed, just note if the baby gags during sucking task	☐	☐

**7. Tongue Tie Screen** [2, 3, 4, 5, 6.] ☐ Not attempted ☐ Unable to complete<br>Use the previous oral motor assessment and the pictures the family sent through to screen for tongue tie to determine if further assessment/management is indicated.			
	**Unable to assess**	**Typical**	**Of concern/needs further investigation**
**Tongue posture during crying**	☐	☐ Elevation of tongue	☐ Elevation of lateral edges of tongue only
**Shape of elevated tongue**	☐	☐ Round or square	☐ Heart or v-shaped
**Tongue lateralisation**	☐	☐ Complete	☐ No lateralisation
**Lingual frenulum**	☐	☐ Visible	☐ Very short/not visible
**Frenulum thickness**	☐	☐ Thin	☐ Thick
**Frenulum attachment to tongue**	☐	☐ Midline	☐ At apex of tongue
**Extension of tongue**	☐	☐ Protrudes past lips	☐ Does not protrude past lips
**Assessment**	☐ WNL	☐ Concerns – recommend further assessment	

8. Non-nutritive suck assessment ☐ Not attempted ☐ Unable to complete

Camera position 3 – Close up view of child's mouth. The parent/carer will need to have both hands free.

Assess the child's sucking on dummy and/or finger.

- Dummy: *Observe the child sucking for a short period then ask the parent to try to remove the dummy from the child's mouth. Ask the parent to describe if the child provides weak or strong resistance (negative pressure)*
- *Finger: Instruct the parent to gently place their little finger (nail side down) into the child's mouth and describe what the sucking feels like. Have the parent count out loud each time they feel a suck to help you get an idea of rhythm. Once you have finished this, ask the parent to try to remove their finger from baby's mouth and describe if the child provides weak or strong resistance (negative pressure).*

Response to stimulus within 3 seconds	☐ Yes	☐ No	
Positive pressure generated (compression)	☐ Yes	☐ No	
Negative pressure generated (suction)	☐ Yes	☐ No	
Rhythmic pattern observed (6:1 – 8:1 suck/swallow ratio)	☐ Yes	☐ No	
Small rhythmic jaw excursions	☐ Yes	☐ No	
Tongue cupping reported/felt	☐ Yes	☐ No	☐ NA

**9. Bottle-feeding assessment** [2, 3]<br>Camera position 4 - Device on 45-degree angle from child with parent/carer in frame			
Ask the parent/carer to feed their child how they usually would			
**Volume offered:**	**Feed time:**		
Set up #1 (bottle type, teat type, position and fluid thickness e.g. thin, slow flow teat, semi-upright):			
Set up #2 (if applicable):			
Set up #3 (if applicable):			
	#1	#2	#3
**Oral Phase**	☐ **WNL**<br>☐ **Difference**	☐ **WNL**<br>☐ **Difference**	☐ **WNL**<br>☐ **Difference**
Nil anticipatory mouth opening			
Inadequate flange			
Poor lip seal			
Impaired milk transfer			
Clicking during feeding			
Gagging			
Tongue thrust			
Anterior fluid loss			
**Suck-swallow**	☐ **WNL**<br>☐ **Difference**	☐ **WNL**<br>☐ **Difference**	☐ **WNL**<br>☐ **Difference**
Uncoordinated suck- swallow breath			
3-6 sucks per swallow			
6+ sucks per swallow			
**Physiological changes**	☐ **WNL**<br>☐ **Difference**	☐ **WNL**<br>☐ **Difference**	☐ **WNL**<br>☐ **Difference**
Sweating			
Color change – red			
Color change – blue			
Hiccups			
Eye tearing			
Sneezing			
Startle			
Twitching			
Tremor			
Gasp			
Sigh			
Fluctuating tone from normal to flaccid			
Excessive diffuse movements			
**Disengagement cues**	☐ **WNL**<br>☐ **Difference**	☐ **WNL**<br>☐ **Difference**	☐ **WNL**<br>☐ **Difference**
Gaze aversion			
Grizzle			
Crying			
Refusal			
Dozing			
Turning away			
Hyperextension of trunk, arms, hands or legs			
Hypertonicity (arching, finger splaying, fisting)			
Shut down response (e.g. falling asleep)			

**Feeder response to cues**	☐ **WNL**<br>☐ **Difference**	☐ **WNL**<br>☐ **Difference**	☐ **WNL**<br>☐ **Difference**
Feeder not recognising and/or responding to infant stress/disengagement cues			
**Respiratory changes**	☐ **WNL**<br>☐ **Difference**	☐ **WNL**<br>☐ **Difference**	☐ **WNL**<br>☐ **Difference**
Wheeze			
Stridor			
Apnoea			
Nasal flaring			
Tracheal tug			
Intercostal recession			
Substernal recession			
Increased respiratory rate			
Laboured/noisy breathing			
**Indicators of penetration +/- aspiration**	☐ **WNL**<br>☐ **Difference**	☐ **WNL**<br>☐ **Difference**	☐ **WNL**<br>☐ **Difference**
Coughing (developmental)			
Coughing (of concern)			
Choking			
Wet vocal quality			
Wet breathing			
Multiple swallows			
**Other**			
Nasal congestion			
Nasal regurgitation			

**10. Assessment summary -** *Complete after observation of feeding*		
Overall feeding skills	☐ WNL	☐ Impaired
Ranking on Functional Oral Intake Scale – Suckle Feeds and Transitional Feeds[3]	☐ 1 – No oral intake (NBM)	
	☐ 2 – Tube dependent with minimal attempts at food or liquids	
	☐ 3- Tube dependent with consistent intake of food or liquids	
	☐ 4- Total oral diet, but requiring special preparation (e.g. foods different texture to those of peers, thickened liquids, liquid supplements) +/- compensations (e.g. special feeding equipment, feeder uses special strategies)	
	☐ 5- Total oral intake without special preparation (e.g. thin liquids, foods same as peers) but with compensations (e.g. special feeding equipment, feeder uses special strategies)	
	☐ 6- Total oral intake with no restrictions relative to peers	
Eating and Drinking Ability Classification System (EDACS) level [7]	☐ I – Eats and drinks safely and efficiently	
	☐ II – Eats and drinks safely but with some limitations to efficiency	
	☐ III – Eats and drinks with some limitations with safety; may be limitations to efficiency	
	☐ IV – Eats and drinks with significant limitations to safety	
	☐ V – Unable to eat or drink safely – tube feeding may be considered to provide nutrition	

**11. Recommendations -** *Complete after observation of feeding*				
**Recommended fluid**	☐ Thin	☐ Slightly thick	☐ Mildly thick	☐ Nil
**Modification to feeding equipment required?**	☐ Yes (please specify below)		☐ No	
	☐ Wide based teat	☐ Standard neck teat	☐ Syringe	
	☐ Slow flow teat	☐ Medium flow teat	☐ Fast flow teat	
	☐ X/Y cut teat	☐ Cleft teat	☐ Special needs feeder	
	☐ Change teat brand	☐ Other (specify)		
**Modification required to positioning?**	☐ Yes (please specify below)	☐ No		
	☐ Swaddled	☐ Upright		
	☐ Side-lying	☐ Semi-upright		
**Feeding strategies required?**	☐ Yes (please specify below)	☐ No		
	☐ Jaw support	☐ Cheek support	☐ Pacing	
	☐ Volume/time limit	☐ Drop tastes		

**12. Session outcome**		
☐ Discharge	☐ Review	
	☐ Urgent	☐ Non-urgent

References:

1. American Speech-Language-Hearing Association. Pediatric Dysphagia. Available online: https://www.asha.org/practice-portal/clinical-topics/pediatric-dysphagia/ (accessed on 30 March 2019).
2. Wolf, L.S.; Glass, R.P. *Feeding and Swallowing Disorders in Infancy: Assessment and Management*; Hammill Institute on Disabilities: Austin, Texas, United States of America, **1992**.
3. Hall, K. *Pediatric Dysphagia Resource Guide*, 1st ed.; Cengage Learning: Boston, MA, USA, 2000.
4. Dodrill, P. Evaluating feeding and swallowing in infants and children. In *Dysphagia: Clinical Management in Adults and Children*, 2nd ed.; Groher, M.E., Crary, M.A., Eds.; Mosby: Maryland Heights, MO, USA, 2016; pp. 205–323.
5. Ballard, J.L.; Auer, C.E.; Khoury, J.C. Ankyloglossia: Assessment, incidence and effect of freunuloplasty on the breastfeeding dyad. *Pediatrics* **2002**, *101*, e63.
6. Martinelli, R.L.C.; Marchesan, I.Q.; Berretin-Felix, G. Lingual frenulum protocol with scores for infants. *Int. J. Orofac. Myol.* **2012**, *38*, 104–112.
7. Sellers, D.; Mandy, A.; Pennington, L.; Hankins, M.; Morris, C. Development and reliability of a system to classify the eating and drinking ability of people with cerebral palsy. *Dev. Med. Child Neurol.* **2014**, *56*, 245–251, doi:10.1111/dmcn.12352.
